# Prediction of Protein Hotspots from Whole Protein Sequences by a Random Projection Ensemble System

**DOI:** 10.3390/ijms18071543

**Published:** 2017-07-18

**Authors:** Jinjian Jiang, Nian Wang, Peng Chen, Chunhou Zheng, Bing Wang

**Affiliations:** 1 School of Electronics and Information Engineering, Anhui University, Hefei 230601, China; jiangjj@aqnu.edu.cn (J.J.); wn_xlb@ahu.edu.cn (N.W.); 2 School of Computer and Information, Anqing Normal University, Anqing 246133, China; 3 Institute of Health Sciences, Anhui University, Hefei 230601, China; 4 School of Electronic Engineering & Automation, Anhui University, Hefei 230601, China; zhengch99@126.com; 5 School of Electrical and Information Engineering, Anhui University of Technology, Ma’anshan 243032, China

**Keywords:** random projection, hot spots, IBk, ensemble system

## Abstract

Hotspot residues are important in the determination of protein-protein interactions, and they always perform specific functions in biological processes. The determination of hotspot residues is by the commonly-used method of alanine scanning mutagenesis experiments, which is always costly and time consuming. To address this issue, computational methods have been developed. Most of them are structure based, i.e., using the information of solved protein structures. However, the number of solved protein structures is extremely less than that of sequences. Moreover, almost all of the predictors identified hotspots from the interfaces of protein complexes, seldom from the whole protein sequences. Therefore, determining hotspots from whole protein sequences by sequence information alone is urgent. To address the issue of hotspot predictions from the whole sequences of proteins, we proposed an ensemble system with random projections using statistical physicochemical properties of amino acids. First, an encoding scheme involving sequence profiles of residues and physicochemical properties from the AAindex1 dataset is developed. Then, the random projection technique was adopted to project the encoding instances into a reduced space. Then, several better random projections were obtained by training an IBk classifier based on the training dataset, which were thus applied to the test dataset. The ensemble of random projection classifiers is therefore obtained. Experimental results showed that although the performance of our method is not good enough for real applications of hotspots, it is very promising in the determination of hotspot residues from whole sequences.

## 1. Introduction

Hotspot residues contribute a large portion of the binding energy of one protein in complex with another protein [1,2], which are always surrounded by residues contributing less binding energy. These are not uniformly distributed for the binding energy of proteins over their interaction surfaces [1]. Hotspots are important in the binding and the stability of protein-protein interactions and thus key to perform specific functions in the protein [3,4]. Actually, hotspots are difficult to determine. A common determination method is the method of alanine scanning mutagenesis experiments, which identify a hotspot if a change in its binding free energy is larger than a predefined threshold when the residue is mutated to alanine. However, this method is costly and time consuming.

Several databases stored experimental and computational hotspot residues and the details of hotspots’ properties. The first database for storing experimental hotspots was the Alanine Scanning Energetics Database (ASEdb) by the use of alanine scanning energetics experiments [5]. Another database is the Binding Interface Database (BID) developed by Fischer et al., which mined the primary scientific literature for detailed data about protein interfaces [6]. These databases are commonly used in previous works on hotspot identification. The Protein-protein Interactions Thermodynamic Database (PINT) is another database that mainly accumulates the thermodynamic data of interacting proteins upon binding along with all of the experimentally-measured thermodynamic data (Kd, Ka, ΔG, ΔH and ΔCp) for wild-type and mutant proteins [7]. It contains 1513 entries in 129 protein–protein complexes from 72 original research articles, where only 33 entries have complete 3D structures deposited in PDB (Protein Data Bank), in the first release of PINT. Recently, Moal et al. built the SKEMPI (Structural Kinetic and Energetic database of Mutant Protein Interactions) that has collected 3047 binding free energy changes from 85 protein-protein complexes from the literature [8].

Although some databases stored hotspot residues, few of the protein complexes were solved. Computational approaches were proposed to identify hotspot residues, and they were complementary to the experimental methods. Some methods predicted hotspots by energy function-based physical models [3,9,10,11], molecular dynamics simulation-based approaches [12,13,14], evolutionary conservation-based methods [4,15,16] and docking-based methods [17,18]. Some methods adopted machine learning methods for the hotspot prediction, such as graph-based approaches [19], neural network [20], decision tree [3,21], SVM (Support Vector Machine) [22], random forest [23] and the consensus of different machine learning methods [24], combining features of solvent accessibility, conservation, sequence profiles and pairing potential [20,23,25,26,27,28,29].

All of the previous methods were developed to identify hotspots from a part of residues in the interface regions. They always worked on selected datasets containing almost the same numbers of hotspots and non-hotspots. The ratio of the number of hotspots to that of residues in whole datasets is around 20∼50%, for example: BID contains 54 hotspots and 58 non-hotspots; 58 hotspots and 91 non-hotspots are in the ASEdb dataset; and SKEMPI contains 196 hotspots and 777 non-hotspots [29]. However, no more than 2% of the residues in whole protein sequences are hotspots. The issue of identifying hotspots from whole protein sequences in our study is more difficult than others, but more interesting.

Most hotspot prediction methods are structure-based, which cannot be applied to protein complexes without the information of protein structures [3,22,23]. Therefore, identifying hotspots from the protein sequence only is important. Moreover, few works identified hotspot residues from the whole protein sequences. To address these issues, here, we propose a method that predicts hotspots from the whole protein sequences using physicochemical characteristics extracted from amino acid sequences. A random projection ensemble classifier system is developed for the hotspot predictions. The system involves an encoding scheme integrating sequence profiles of residues and the statistical physicochemical properties of amino acids from the AAindex1 (Amino Acid index1 database) dataset. Then, the random projection technique was adopted to obtain a reduced input space, but to retain the structure of the original space. Several better classifiers with the IBk algorithm are obtained after the use of random projections. The ensemble of good classifiers is therefore constructed. Experimental results showed that our method performs well in hotspot predictions for the whole protein sequences.

## 2. Results

### 2.1. Performance of the Hotspot Prediction

In the running of the random projection-based classifier, different random projections in Equation (1) construct different classifiers. After running the classifier 100 times, 100 classifiers with random projections *R* are formed and trained on the training subset $D_{tr}^{k}$. As a result, 100 predictions are obtained. All of the classifiers are ranked in terms of the prediction measure *F1*. The ensemble of several top *N* classifiers is then tested on the test subset $D_{ts}^{k}$. In this work, the ASEdb0 is regarded as the training dataset, and the test dataset is BID0; while the predictions on the ASEdb0 dataset are also tested by training on the BID0 dataset.

Table 1 shows the performance of the top individual classifiers trained by the ASEdb0 dataset and the prediction performance on the BID0 dataset. The individual classifiers are ranked in terms of the *F1* measure in the training process. The top classifiers yield good predictions on the BID0 dataset. It achieves an F1 of 0.109, as well as a precision of 0.069 at a sensitivity of 0.259 in the training process and, therefore, yields an F1 of 0.315, as well as a precision of 0.220 at a sensitivity of 0.558 in the test process. Here, the dimensionality of the original data is reduced from 7072 to only five.

Table 2 shows the performance comparison of the ensembles of the top *N* classifiers. In the classifier ensemble, the majority vote technique was applied to the ensemble, i.e., one residue will be identified as the hotspot if half of the *N* classifiers predict it to be the hotspot. Here, seven ensembles of the number of top classifiers are listed, i.e., 2, 3, 5, 10, 15, 25 and 50. From Table 2, it can be seen that the ensemble of the top three classifiers with the majority vote yields a good performance compared with other classifier ensembles. It yields an *MCC* (Matthews Correlation Coefficient) of 0.428, as well as a precision of 0.245 at a sensitivity of 0.793, for testing on the ASEdb0 dataset by training on the BID0 dataset; and it yields an *MCC* of 0.601, as well as a precision of 0.440 at a sensitivity of 0.846, for testing on the BID0 dataset by training on the ASEdb0 dataset. The ensemble of the top three classifiers resulted in a dramatic improvement, compared with the top three individual classifiers. The reason for the improvement is most likely in that a suitable random projection makes the classifier more diverse, where the detailed results are not shown here. Previous methods also showed that the ensemble of more diverse classifiers yielded more efficient predictions [30].

It seems that the more top classifiers the ensemble contains, the worse the ensemble performs. The ensemble with the top 50 classifiers performs the worst both for testing on the ASEdb0 and the BID0 dataset. Therefore, a suitable number of top classifiers can improve the predictions of hotspot residues. Moreover, our method on the BID0 dataset performs better than that on the ASEdb0 dataset, maybe because of the larger ratio of hotspots to the total residues in BID0 (1.831%) than that in ASEdb0 (1.445%).

Furthermore, the performance comparison of ensembles with different numbers of reduced instance dimensions by the random projection technique was investigated. The ensembles of random projections with seven reduced dimensions were built, i.e., the dimensions of 1, 2, 5, 10, 20, 50 and 100. The ensemble with the reduced dimension of five performs the best among the seven ensembles, while the ensemble of the top three classifiers with instance dimension of one also performs well in the hotspot predictions for the whole sequences of proteins, which yields an *MCC* of 0.475, as well as a precision of 0.704 at a sensitivity of 0.328. Table 3 shows the performance comparison of the classifier ensemble with different numbers of reduced dimensions on the BID0 test dataset.

**Figure 1:**
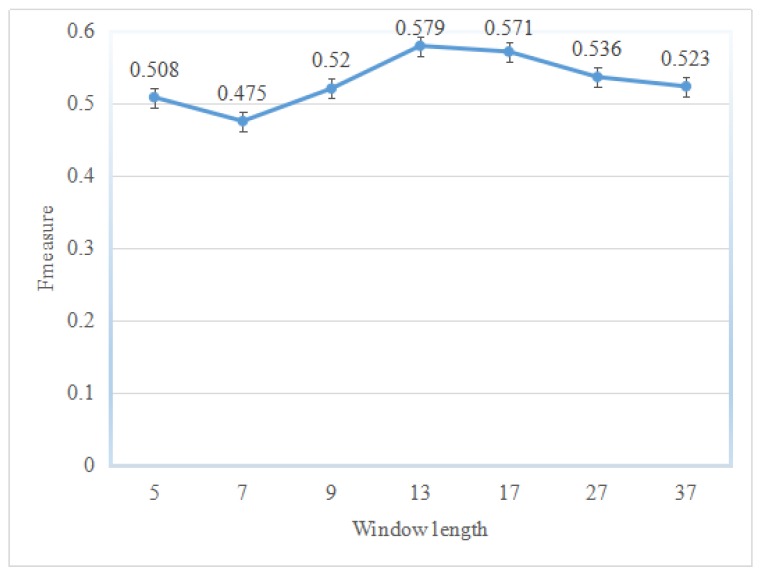
Prediction performance for different sliding windows in instance encoding on the BID0 dataset training by the ASEdb0 dataset. The symbol “I” for each window denotes the calculation error of prediction performance in *F1*.

This study adopted the window length technique to encode input instances of classifiers; however, the sliding window technique makes the performance of the classifier varied. To show which window length makes the classifiers better for a specific type of dataset, several windows with different lengths were investigated. Figure 1 shows the prediction performance on different sliding windows on the BID0 dataset. Among the seven sliding windows, the window with length 13 performs the best, which yields an *F1* of 0.579. It should be mentioned here that classifier ensembles with a suitable window length perform better than those with a smaller or bigger length.

### 2.2. Comparison with Other Methods

So far, few works identified hotspots from the whole protein sequences by sequence information alone. Some top hotspot predictors did the predictions based on protein structures. Most of hotspot prediction methods predicted hotspots from protein-protein interfaces or from some benchmark datasets, such as ASEdb0 and BID0, which contained approximately the same hotspots and non-hotspots. Therefore, the random predictor is used to compare with our method. The random predictor was run 100 times, and the average performance was calculated. Furthermore, for prediction comparison, the tool of ISIS (Interaction Sites Identified from Sequence) [20] on the PredictProtein server [31] was adopted, which has been applied in hotspot predictions on the dataset of interface residues [20]. ISIS is a machine learning-based method that identified interacting residues from the sequence alone. Similar to our method, although the method was developed using transient protein-protein interfaces from complexes of experimentally-known 3D structures, it only used the sequence and predicted 2D information. In PredictProtein, it predicted a residue as a hotspot if the prediction score of the residue was bigger than 21, otherwise being non-hotspot residues. Since PredictProtein currently cannot process short input sequences less than 17 residues, protein sequences in PDB names “1DDMB” and “2NMBB” were removed from the BID0 test set. We tested all of the sequences of more than 17 residues on the BID0 dataset, and the performance of hotspot predictions on the dataset was obtained. The predictions of ISIS method can be referred to the Supplementary Materials.

Table 4 lists the hotspot prediction comparison in detail. Our method developed a random projection ensemble system yielding a final precision of 0.440 at a sensitivity of 0.846 by the use of sequence information only. Results showed that our method outperforms the random predictor. Furthermore, our method outperformed the ISIS method. Actually, ISIS was developed to identify protein-protein interactions. The power of ISIS for the identification of hotspot residues was poor. It can predict nine of 47 real hotspots correctly; however, 2920 non-hotspots were predicted to be hotspots in the BID0 dataset.

**Figure 2:**
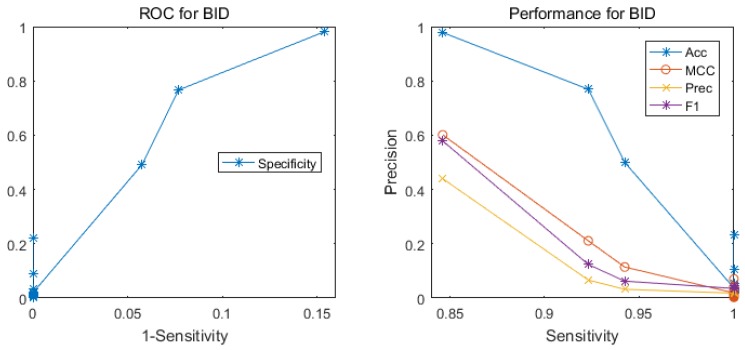
The performance of our method for testing on the BID0 dataset by training on the ASEdb0 dataset. The left graph illustrates the ROC (receiver operating characteristic) curve, and the right one shows the four measure curves with respect to sensitivity.

We also show the performance of classifier ensemble in several measures based on the measure of sensitivity. Figure 2 illustrates the performance of the ensemble classifier with the majority vote for the test set BID0. Although it is very difficult to identify hotspots from the whole protein sequences, our method yields a good result based on sequence information only.

### 2.3. Case Study of Hotspot Predictions

**Figure 3:**
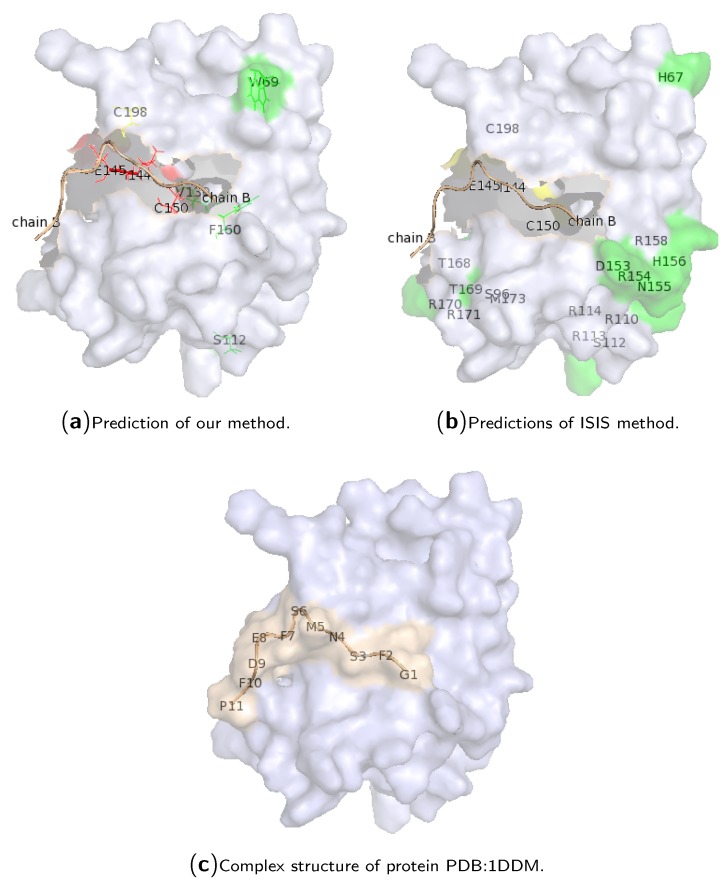
Case study for the complex of protein PDB:1DDM. The subgraphs (**a**,**b**) are shown for the prediction comparison of our method and the ISIS method, respectively, where the chain B of protein 1DDM is colored in wheat. The subgraph (**c**) illustrates the cartoon structure of the protein complex, where the chain B of protein 1DDM is colored in green. Here, red residues are the hotspots that are predicted correctly; green residues are non-hotspots that are predicted to be hotspots; while yellow ones are real hotspots that are predicted to be non-hotspot residues. All other residues are correctly predicted as non-hotspots.

To show the performance of our method on a single protein chain, hotspot predictions for chain “A” of protein PDB:1DDM are illustrated in Figure 3. Protein 1DDM is an in vivo complex containing a phosphotyrosine-binding (PTB) domain (chain “A”) of the cell fate determinant Numb, which can bind a diverse array of peptide sequences in vitro, and a peptide containing an amino acid sequence “NMSF” derived from the Numb-associated kinase (Nak) (chain “B”). The Numb PTB domain is in complex with the Nak peptide. The chain “A” contains 135 amino acid residues, where only residues E144, I145, C150 and C198 are real hotspot residues in complex with the chain “B” of the protein (which contains 11 amino acid residues; see Figure 3c). Our method correctly predicted the first three true hotspots, and hotspot residue 198 was predicted as a non-hotspot, while residues 69, 112, 130 and 160 were wrongly predicted as hotspot residues. All of them are located at the surface of the protein structure. The results of ISIS are also illustrated in Figure 3b. The ISIS method cannot identify the four true hotspot residues, although most of the hotspot predictions are located at the surface of the protein.

## 3. Materials and Methods

### 3.1. Hot Spot Definitions

As we know, a residue is defined as a hotspot by the change of the binding free energy (ΔΔG) higher than a threshold, if mutated to alanine. Several thresholds were adopted in previous works. Many works defined residues as hotspots when their ΔΔGs are higher than 2.0 kcal/mol, and other residues with ΔΔG from 0–2.0 kcal/mol were defined as non-hotspots [21,22,23]. Ofran et al. used another definition that defined residues with ΔΔG above 2.5 kcal/mol as hotspots and those with ΔΔG= 0 kcal/mol (i.e., no change in binding energy) as non-hotspots [20]. Moreover, Tuncbag and colleagues defined hotspots as those with ΔΔG higher than 2.0 kcal/mol and non-hotspots as those with ΔΔG from 0–0.4 kcal/mol [24]. Previous works also investigated several definitions of hotspots [26,29]. They concluded that different definitions of hotspots and non-hotspots yield different ratios of the number of hotspots to that of non-hotspots and, therefore, change the performances of hotspot prediction methods [26,29]. In this paper, residues higher than 2.0 kcal/mol are defined as hotspots and all other residues in the whole protein sequences as non-hotspots, no matter if their position is in interfaces, surfaces or any other regions.

### 3.2. Datasets

Since this work addresses the issue of hotspot residue predictions for the whole sequences of proteins, the definitions of hotspot residues are the same as those of the ASEdb and BID datasets, while all of the other residues in the protein sequences are considered as non-hotspot residues.

Two commonly-used benchmark datasets are used in this work. The first dataset is ASEdb [5]. To clean the proteins in ASEdb, protein sequences in the dataset were removed when the sequence identity between any two sequences was higher than 35%. Based on the hotspot definition in this study, we constructed a new ASEdb0 dataset consisting of 58 hotspots from the ASEdb dataset and 3957 non-hotspots of the other residues in whole protein sequences, totaling 4015 residues in our new ASEdb0 dataset.

The BID dataset [6] is the other one used in this work. The dataset was filtered in the same manner as the ASEdb dataset. As a result, we constructed a new BID0 dataset consisting of 54 hotspots from the BID dataset and 2895 non-hotspots from the rest of the residues in the whole protein sequences, totaling 2949 residues in our new BID0 dataset. The data in the two datasets came from different complexes and were mutually exclusive. Table 5 lists the composition of hotspots and non-hotspots.

### 3.3. Feature Encoding Scheme

The AAindex1 database [32] contained 544 numerical indices representing various physicochemical and biochemical properties of amino acids. It collected published indices with a set of 20 numerical values representing different properties of amino acids. It also contained the results of cluster analysis using the correlation coefficient as the distance between two indices. All data were derived from published literature.

The protein sequence profile of one amino acid is a set of 20 numerical values representing the evolution of the amino acid residue, where each value represents the frequency by which residue was mutated into another amino acid residue. It can be used to recognizing remote homologs and plays an important role in protein sequence database search, protein structure/function prediction and phylogenetic analysis. Protein sequence profiles are always obtained by a BLAST (Basic Local Alignment Search Tool) program, such as the commonly-used program of PSI-BLAST (Position-Specific Iterative Basic Local Alignment Search Tool) [33]. Therefore, for the residue $R_{i}$ of one protein sequence, the multiplication $MSK_{i}^{j}$ of the sequence profile $SP_{i}$ of residue $R_{i}$ and one physicochemical amino acid property $AAP^{j}$ can represent the statistical evolution of the amino acid property [34,35,36], i.e., $MSK_{i}^{j}=SP_{i}\times AAP^{j}$, where $SP_{i}$ and $AAP^{j}$ are both vectors of 1×20. The multiplication for residue $R_{i}$ results in a set of 20 numerical vectors $MSK_{i}^{j}$. The standard deviation $STD_{i}^{j}$ of the multiplication is then obtained. For residue $R_{i}$, the 544 amino acid AAindex1 properties yield a set of 544 standard deviations $STD_{i}=STD_{i}^{j},j=1544$, which will be used for encoding residue $R_{i}$. Our previous work has shown that the standard deviations of the multiplications can reflect the evolutionary variance of the residue $R_{i}$ along with the amino acid property $AAP^{j}$ [29,34,35].

To encode the residue $R_{i}$ in one protein sequence, a sliding window involving residues centered at the residue $R_{i}$ is considered, i.e., several neighboring residues are used to represent the center residue $R_{i}$. Therefore, a set of winLen×544=7072 numerical values represents the residue $R_{i}$, where winLen=13 is the sliding window length in this work. A similar vector representation can be found in our previous work [29,34,35]. For the residue $R_{i}$, it is represented by a 1×7072 vector $V_{i}$, whose corresponding target value $T_{i}$ is 1 or 0, denoting whether the residue is a hotspot or not. Therefore, our method is developed to learn the relationship between input vectors *V* and the corresponding target array *T* and tries to make its output Y=f(V) as close to the target *T* as possible.

### 3.4. IBk Classifier Ensemble by the Random Mapping Technique

The random projection technique can be traced back to the work done by Ritter and Kohonen [37], which reduced the dimensionality of the representations of the word contexts by replacing each dimension of the original space by a random direction in a smaller-dimensional space. From the literature [37,38], it seems surprising that random mapping can reduce the dimensionality of the data in a manner that preserves enough structure of the original dataset to be useful. Kaski used both analytical and empirical evidence to explain the reason why the random mapping method worked well in high-dimensional spaces [39].

Given the original data, $X\in {ℜ}^{N\times L1}$, let the linear random projection be the multiplication of the original instances by a random matrix $R\in {ℜ}^{L1\times L2}$, where the element in the matrix ranges from 0–1. The matrix *R* is composed of random elements, and each column has been normalized to unity. The projection:

(1) $$\begin{array}{c} X^{R}=XR=\sum_{i}\left(x_{i}\times r_{i}\right) \end{array}$$

yields a dimensionality-reduced instance $X^{R}\in {ℜ}^{N\times L2}$ from dimension L1 to L2, where $x_{i}$ is the *i*-th sample of the original data, $r_{i}$ is the *i*-th column of the random matrix and L2≪L1. In Equation (1), each original instance with dimension L1 has been replaced by a random, non-orthogonal direction L2 in the reduced-dimensional space [39]. Therefore, the dimensionality of the original instance is reduced from 7072 to a rather small value.

The dimension-reduced instances are then input into the classifier with the IBk algorithm. The IBk algorithm, implementing the k-nearest neighbor algorithm, is a type of instance-based learning, where the function is only approximated locally, and all computations are deferred until classification. The simplest of the IBk algorithms among machine learning algorithms was adopted since we want to ensemble diverse classifiers and expect to yield good results. Previous results showed that the generalization error caused by one classifier can be compensated by other classifiers; therefore, the ensemble of some diverse classifiers can yield significant improvement [40].

In the hotspot prediction, the multiplication of the *k*-th random projection $R_{k}$ on the original instances (X,Y) forms a set of instances $D^{k}=\left\{\left(X_{i}^{R_{k}},Y_{i}\right)\right\},\;i=1,\ldots ,N$, where *N* and *K* denote the number of training instances and that of random projections, respectively. For the *k*-th random projection, the instances $D^{k}$ are generated from the original instances (X,Y) as an input to an IBk classifier, and thus, it forms a classifier $IBk_{k}\left(x\right)$, where *x* is a training instance. To train the classifier $IBk_{k}\left(x\right)$, the instance set $D^{k}$ is divided into training dataset $D_{tr}^{k}$ and test dataset $D_{ts}^{k}$ by 10-fold cross-validation. For training the classifier, the training dataset $D_{tr}^{k}$ is divided into training subset $D_{tr}^{k-tr}$ and test subset $D_{tr}^{k-ts}$ again. The training process retains the top classifiers on some random projections, and in the test process, they are applied to test the test dataset $D_{ts}^{k}$.

After running random projection 100 times, top classifiers in the *F1* measure are retained for testing the test dataset $D_{ts}^{k}$. The ensemble of top classifiers yields the final predictions. The mjority vote technique was always used in classifier ensemble and often made a dramatic improvement [41]. Here, a residue is predicted as a hotspot if half of the classifiers identified it as positive Class 1, otherwise it is a non-hotspot residue.

**Figure 4:**
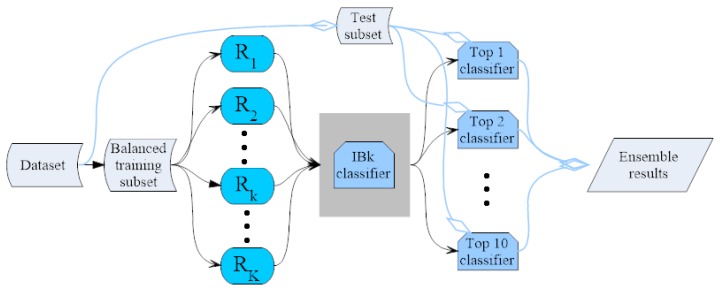
The flowchart of the ensemble system for the hotspot prediction. Here, $R_{k}$ means the *k*-th random projection. The IBk implements k-Nearest Neighbors (KNN) algorithm. Here the black arrows denote the flow of the training subset, while the blue ones are that of the test subset.

Moreover, since the hotspot dataset is extremely imbalanced, containing only 1.4% of hotspots, balancing the dataset is necessary to avoid the overfitting of the classifier. Therefor, the training dataset $D_{tr}^{k-tr}$ is resampled and then consists of positive instances and negative instances with roughly the same number. The ensemble system can be seen i Figure 4.

### 3.5. Hot Spot Prediction Evaluation

To evaluate hotspot predictions, in this work, we adopted four evaluation measures to show the ability of our model objectively. They are the criteria of sensitivity (*Sen*), precision (*Prec*), F-measure (*F1*) and Matthews correlation coefficient (*MCC*) [34,42] and shown below:

(2) $$\begin{array}{c} Sen=\frac{TP}{TP+FN},\;Prec=\frac{TP}{TP+FP}\\ F1=2\times \frac{Prec\times Sen}{Prec+Sen}\\ MCC=\frac{TP\times TN-FP\times FN}{\sqrt{\left(TP+FN)(TP+FP)(TN+FP)(TN+FN\right)}}, \end{array}$$

where *TP* (true positive) is the number of correctly-predicted hotspot residues; *FP* (false positive) is the number of false positives (incorrectly over-predicted non-hotspot residues); *TN* (true negative) is the number of correctly-predicted non-hotspot residues; and *FN* (false negative) is false negative, i.e., incorrectly under-predicted hotspot residues.

## 4. Conclusions

This paper proposes an ensemble method based on the random projection technique that predicts hotspots from the whole sequences of proteins, using physicochemical characteristics of amino acids. The classifier system involves an encoding scheme integrating sequence profiles of residues and statistical physicochemical properties of amino acids from the AAindex1 dataset. Then, the random projection technique was adopted to obtain a reduced input space for the original input instances, but retaining the structure of the original space. Several top classifiers are obtained after the use of random projections. The ensemble of the top classifiers is therefore constructed. The classifier with random projection ran 50 times, and 50 classifiers were sorted in the *F1* measure in the training step. Applying the 50 classifiers to the test dataset yielded the final hotspot predictions. Results showed that the ensemble of the top three classifiers yields better performance in hotspot predictions. Moreover, random projections with different reduced dimensions were investigated, and the projection with the dimension of five performs the best. To select the most effective sliding window, several sliding windows were investigated for encoding instances, and a window with a length of 13 was chosen finally, which performed the best among the eight windows. It is suggested that our method is promising in computational hotspot prediction for the whole protein sequence.

## Figures and Tables

**Table 1:** Prediction performance of individual classifiers with the reduced dimension of 5 on the Binding Interface Database 0 (BID0) test dataset training by Alanine Scanning Energetics Database 0 (ASEdb) dataset. There are 50 top individual classifiers listed here for a simple comparison between classifiers. Here measures of “*Sen*”, “*Prec*”, “*F1*” and “*MCC*” denote Sensitivity, Precision, F-Measure, and Matthews Correlation Coefficient, respectively.

No.	Training				Test			
	*Sen*	*MCC*	*Prec*	*F1*	*Sen*	*MCC*	*Prec*	*F1*
1	0.259	0.110	0.069	0.109	0.558	0.332	0.220	0.315
2	0.069	0.125	0.250	0.108	0.558	0.357	0.250	0.345
3	0.138	0.080	0.070	0.093	0.212	0.141	0.122	0.155
4	0.069	0.085	0.129	0.090	0.500	0.274	0.173	0.257
5	0.121	0.075	0.071	0.089	0.308	0.194	0.150	0.201
6	0.069	0.083	0.125	0.089	0.096	0.040	0.044	0.060
7	0.069	0.076	0.108	0.084	0.269	0.136	0.096	0.141
8	0.069	0.076	0.108	0.084	0.269	0.129	0.090	0.135
9	0.138	0.071	0.061	0.084	0.558	0.364	0.259	0.354
10	0.138	0.069	0.058	0.082	0.346	0.226	0.173	0.231
11	0.069	0.071	0.098	0.081	0.135	0.038	0.037	0.058
12	0.086	0.066	0.075	0.080	0.615	0.337	0.205	0.308
13	0.052	0.080	0.150	0.077	0.577	0.317	0.196	0.293
14	0.052	0.076	0.136	0.075	0.404	0.227	0.153	0.222
15	0.069	0.064	0.083	0.075	0.135	0.082	0.080	0.100
16	0.052	0.074	0.130	0.074	0.577	0.323	0.203	0.300
17	0.052	0.074	0.130	0.074	0.596	0.279	0.153	0.243
18	0.069	0.062	0.080	0.074	0.404	0.225	0.151	0.220
19	0.069	0.062	0.080	0.074	0.308	0.152	0.102	0.153
20	0.052	0.072	0.125	0.073	0.115	0.030	0.033	0.052
21	0.121	0.058	0.052	0.073	0.192	0.135	0.123	0.150
22	0.052	0.067	0.111	0.071	0.288	0.150	0.105	0.154
23	0.190	0.064	0.044	0.071	0.577	0.281	0.159	0.249
24	0.069	0.056	0.070	0.070	0.269	0.145	0.105	0.151
25	0.086	0.054	0.057	0.069	0.423	0.171	0.095	0.155
26	0.086	0.053	0.057	0.068	0.212	0.079	0.057	0.090
27	0.086	0.051	0.054	0.066	0.365	0.218	0.156	0.218
28	0.052	0.058	0.091	0.066	0.250	0.091	0.060	0.097
29	0.052	0.057	0.088	0.065	0.481	0.237	0.141	0.218
30	0.034	0.095	0.286	0.062	0.519	0.241	0.136	0.215
31	0.034	0.095	0.286	0.062	0.346	0.204	0.146	0.206
32	0.052	0.050	0.073	0.061	0.173	0.095	0.081	0.110
33	0.138	0.048	0.039	0.061	0.442	0.271	0.190	0.266
34	0.052	0.049	0.071	0.060	0.231	0.115	0.085	0.124
35	0.224	0.055	0.035	0.060	0.346	0.186	0.127	0.186
36	0.034	0.078	0.200	0.059	0.250	0.161	0.131	0.172
37	0.207	0.052	0.034	0.059	0.519	0.273	0.167	0.252
38	0.034	0.074	0.182	0.058	0.365	0.238	0.181	0.242
39	0.034	0.064	0.143	0.056	0.192	0.083	0.064	0.096
40	0.052	0.044	0.061	0.056	0.231	0.146	0.120	0.158
41	0.052	0.042	0.059	0.055	0.135	0.070	0.065	0.088
42	0.103	0.038	0.036	0.054	0.327	0.145	0.091	0.143
43	0.103	0.037	0.036	0.053	0.192	0.111	0.093	0.125
44	0.034	0.049	0.095	0.051	0.077	0.013	0.025	0.037
45	0.069	0.035	0.040	0.051	0.154	0.054	0.046	0.071
46	0.121	0.034	0.031	0.050	0.423	0.231	0.151	0.222
47	0.224	0.041	0.028	0.050	0.288	0.172	0.129	0.179
48	0.241	0.037	0.026	0.046	0.308	0.152	0.102	0.153
49	0.052	0.030	0.040	0.045	0.442	0.210	0.125	0.195
50	0.155	0.031	0.026	0.045	0.462	0.252	0.162	0.240

**Table 2:** Prediction performance of the ensemble of the top *N* classifiers with reduced instance dimension of 5 on the two datasets.

Test Set	No. Dimension	*Sen*	*MCC*	*Prec*	*F1*
ASEdb0	2	0.224	0.322	0.481	0.306
	3	0.793	0.428	0.245	0.374
	5	0.897	0.383	0.177	0.295
	10	1.000	0.299	0.103	0.186
	15	1.000	0.219	0.062	0.116
	25	1.000	0.149	0.036	0.070
	50	1.000	0.081	0.021	0.041
BID0	2	0.385	0.260	0.200	0.263
	3	0.846	0.601	0.440	0.579
	5	1.000	0.461	0.226	0.369
	10	1.000	0.283	0.096	0.175
	15	1.000	0.222	0.066	0.124
	25	1.000	0.145	0.038	0.074
	50	1.000	0.078	0.024	0.046

**Table 3:** Prediction performance of the ensemble of the top 3 classifiers with different reduced instance dimensions on the BID0 test dataset.

No. Dimension	*Sen*	*MCC*	*Prec*	*F1*
1	0.328	0.475	0.704	0.447
2	0.328	0.352	0.396	0.358
5	0.846	0.601	0.440	0.579
10	0.846	0.499	0.310	0.454
20	0.481	0.240	0.144	0.221
50	0.500	0.274	0.173	0.257
100	0.538	0.252	0.141	0.224

**Table 4:** Performance comparison of the three methods on the BID0 dataset by training on the ASEdb0 dataset.

Method	Type	*Sen*	*MCC*	*Prec*	*F1*
Our Method	Random Projection	0.846	0.601	0.440	0.579
ISIS	Neural Networks	0.191	0.030	0.026	0.046
Random Predictor		0.983	0.000	0.018	0.035

**Table 5:** The details of the hotspot datasets.

Dataset	Hot Spots	Non-Hotspots	Total Residues	Ratio ${}^{§}$
BID0	54	2895	2949	1.831%
ASEdb0	58	3957	4015	1.445%
BID	54	58	112	48.214%
ASEdb	58	91	149	38.926%

${}^{§}$ The ratio of the number of hotspots to that of total residues in the dataset.

## Supplementary Materials

Supplementary materials can be found at www.mdpi.com/1422-0067/18/7/1543/s1.

## Abbreviations

kNN	k-Nearest Neighbor
*Sen*	Sensitivity
*Prec*	Precision
*F1*	F-Measure
*MCC*	Matthews Correlation Coefficient
ASEdb	Alanine Scanning Energetics Database
BID	Binding Interface Database
SKEMPI	Structural Kinetic and Energetic Database of Mutant Protein Interactions
